# Increased Noise Levels Have Different Impacts on the Anti-Predator Behaviour of Two Sympatric Fish Species

**DOI:** 10.1371/journal.pone.0102946

**Published:** 2014-07-24

**Authors:** Irene K. Voellmy, Julia Purser, Stephen D. Simpson, Andrew N. Radford

**Affiliations:** 1 School of Biological Sciences, University of Bristol, Bristol, United Kingdom; 2 Biosciences, College of Life and Environmental Sciences, University of Exeter, Exeter, United Kingdom; Institute of Marine Research, Norway

## Abstract

Animals must avoid predation to survive and reproduce, and there is increasing evidence that man-made (anthropogenic) factors can influence predator−prey relationships. Anthropogenic noise has been shown to have a variety of effects on many species, but work investigating the impact on anti-predator behaviour is rare. In this laboratory study, we examined how additional noise (playback of field recordings of a ship passing through a harbour), compared with control conditions (playback of recordings from the same harbours without ship noise), affected responses to a visual predatory stimulus. We compared the anti-predator behaviour of two sympatric fish species, the three-spined stickleback (*Gasterosteus aculeatus*) and the European minnow (*Phoxinus phoxinus*), which share similar feeding and predator ecologies, but differ in their body armour. Effects of additional-noise playbacks differed between species: sticklebacks responded significantly more quickly to the visual predatory stimulus during additional-noise playbacks than during control conditions, while minnows exhibited no significant change in their response latency. Our results suggest that elevated noise levels have the potential to affect anti-predator behaviour of different species in different ways. Future field-based experiments are needed to confirm whether this effect and the interspecific difference exist in relation to real-world noise sources, and to determine survival and population consequences.

## Introduction

Noise-generating human activities, including transportation, urban development and resource exploitation, have changed the acoustic environment of many terrestrial and aquatic habitats around the world [1], [2]. Increasing evidence suggests that anthropogenic (man-made) noise can affect the behaviour of a diverse range of animals [2], [3]. However, research has focused primarily on behaviours such as acoustic communication and movement patterns that are difficult to translate into ultimate fitness consequences [3], [4]. Avoidance of predation is crucial if animals are to survive and reproduce successfully [5], yet few studies have investigated the potential impact of anthropogenic noise on anti-predator behaviour (but see [6]–[8]).

It is likely that susceptibility to elevated noise levels will depend on, for instance, species-specific hearing abilities [9], [10] and physiological stress responses [11]; the effect on anti-predator behaviour might also depend on the particular defence strategies employed [5]. However, studies exploring the effect of anthropogenic noise have generally collected data on only a single species (but see [12]–[14]). Since interspecific differences may alter the relative success of each species under conditions of anthropogenic disturbance, experimental tests of responses to the same noise source are important for an understanding of the potential effects on community composition and structure [15].

In water, sound propagates about five times further than in air, whereas light attenuates much faster [16]. Thus, sound plays a particularly important role in the transmission of information and increased noise levels caused by anthropogenic activities, such as seismic measurements, pile-driving, ship traffic and renewable energy operations, may substantially affect aquatic organisms [2]. Many fishes use and produce sounds [17], and there is increasing evidence that at least some species are negatively impacted by anthropogenic noise [2], [16], [18]. However, there has been little consideration of fish behaviours that directly affect fitness [8]. There is wide variation among fish species in hearing ability [19], [20], in sensitivity to stress [21] and in anti-predator defences [22]. In the latter case, for instance, species possessing body armour stay longer in potentially dangerous locations, initiate escape behaviour later, at shorter flight distances and hide less often for shorter time periods than unarmoured species [23]–[25]. Thus, there are strong reasons to expect interspecific differences in the response to noise [26], [27].

In our laboratory study, we investigated whether and how additional noise might impact the anti-predator responses of two sympatric fish species – the three-spined stickleback (*Gasterosteus aculeatus*) and the European minnow (*Phoxinus phoxinus*). We conducted our experimental work in captive conditions to allow careful control of potential confounding factors and the collection of detailed behavioural data (see also [7], [14], [28], [29]). Playbacks in tanks generate complex sound fields and noise profiles that are unlikely to match closely the original source [30], [31]. Such set-ups also generate high levels of particle motion (all fish detect this element of sound; [32]), so results pertain directly only to the near-field. However, our aim was to provide an initial exploration of how increased noise levels might affect behaviour essential for survival, a topic that has received very little previous empirical attention. Our approach therefore parallels the early work on other environmental stressors, such as ocean acidification and global warming, where laboratory studies were used to provide a valuable starting point in our understanding of potential impact while accepting that the “stressor experience” does not fully replicate real-world conditions [33], [34].

Three-spined sticklebacks inhabit a wide variety of freshwater, brackish seashore and estuarine areas [35], [36], and thus encounter anthropogenic noise emitted from such sources as boats, ship traffic, and pile-driving. Their abundance and the wide range of taxa that prey on them (including invertebrates, reptiles, mammals, fish and birds; reviewed in [37], [38]) mean sticklebacks play an important role in aquatic ecosystems. Moreover, they are a model species used in laboratories all over the world in many different research fields [39]–[41]. Minnows can co-occur with sticklebacks, are similar in size and diet, and are vulnerable to the same guild of predators [35], [42], [43]. However, unlike sticklebacks [44], minnows do not possess body armour [45], which is likely to influence their relative levels of risk-taking behaviour [23]–[25]). The two species potentially also differ in their hearing capabilities (see [46], [47]): minnows probably have more sensitive hearing than sticklebacks, with behavioural responses reported to tones up to 5 kHz in minnows [46], whereas hearing sensitivities of nine-spined sticklebacks (*Pungitius pungitius*), a species closely related to three-spined sticklebacks, were reported to decline from frequencies of 400 Hz and higher [47]. The questioning of methods used to assess hearing in fish and the variability between laboratories means, though, that definite conclusions based on the available data are not possible at this time [20], [48].

In our experiment, we explored anti-predator behaviour in response to an overhead visual stimulus (a seagull model that moved over the top of the tank) when fish were exposed to additional noise (playback of field recordings of a ship passing through a harbour) compared with control conditions (playback of recordings from the same harbours without ship noise). When attacked by a diving piscivorous bird such as a seagull [49], fish respond with a range of behaviours including freezing, escape attempts or movement to shelter [50]. We hypothesised that, if additional noise causes a stress response triggering reduced activity and locomotion [51], [52], acts as a distraction or masks an important acoustic cue (see [7], [14]), individuals might be less likely to respond or to respond more slowly to the predatory stimulus. However, if additional noise results in stress responses triggering greater arousal or alertness [52], or increased vigilance to compensate for any masking of acoustic information (see [7], [14]), threats might be more likely to be detected or detected sooner. Previous work on the effects of increased noise has demonstrated that the resultant reduction in food intake in the two study species is underpinned by different mechanisms [14]. Since unarmoured minnows are likely to be more risk-averse than sticklebacks, and potentially have better hearing, we also predicted interspecific differences in how additional noise affects anti-predator behaviour.

## Materials and Methods

### Ethics Statement

All procedures complied with the Association for the Study of Animal Behaviour and Animal Behaviour Society Guidelines for the Use of Animals in Research and were accredited by the University of Bristol Ethical Committee (University Investigator Number: UB/10/034, see also Voellmy et al. [14]). Fish were only tested after acclimatisation to the test setup (i.e. when they did not hide or stop moving for longer than 3 s in the test tank prior to trials). Moreover, fish showed only brief startles or short cessations of movements in response to playbacks of additional noise, and those which startled or stopped their activity resumed pre-trial activity levels within minutes after playbacks ended. At the end of each trial, all fish resumed normal pre-experimental behaviour in their holding tanks.

### Study animals and holding conditions

Thirty-nine three-spined sticklebacks (35 as focal fish, 4 as social companions necessary to facilitate normal behaviour of social fish in experimental conditions; pers. obs.), and thirty-one European minnows (27 focal fish, 4 companions) were sourced from wild origin (wild-caught with Environment Agency permissions, or from commercial suppliers with known wild origin). Each species was housed separately in groups of up to 20 sticklebacks and up to 12 minnows in 100 litre laboratory glass tanks containing artificial plants; for minnows, sand substrate and half-flower pots for supplementary shelter were added. Holding conditions replicated non-breeding conditions appropriate to each species. All capture, holding tank and husbandry details, including acoustic conditions of holding tanks, are the same as in Voellmy et al. [14]. All fish used in this study were healthy, non-breeding adults of unknown sex and were naïve to the test procedure.

### Playback treatments

**Figure 1:**
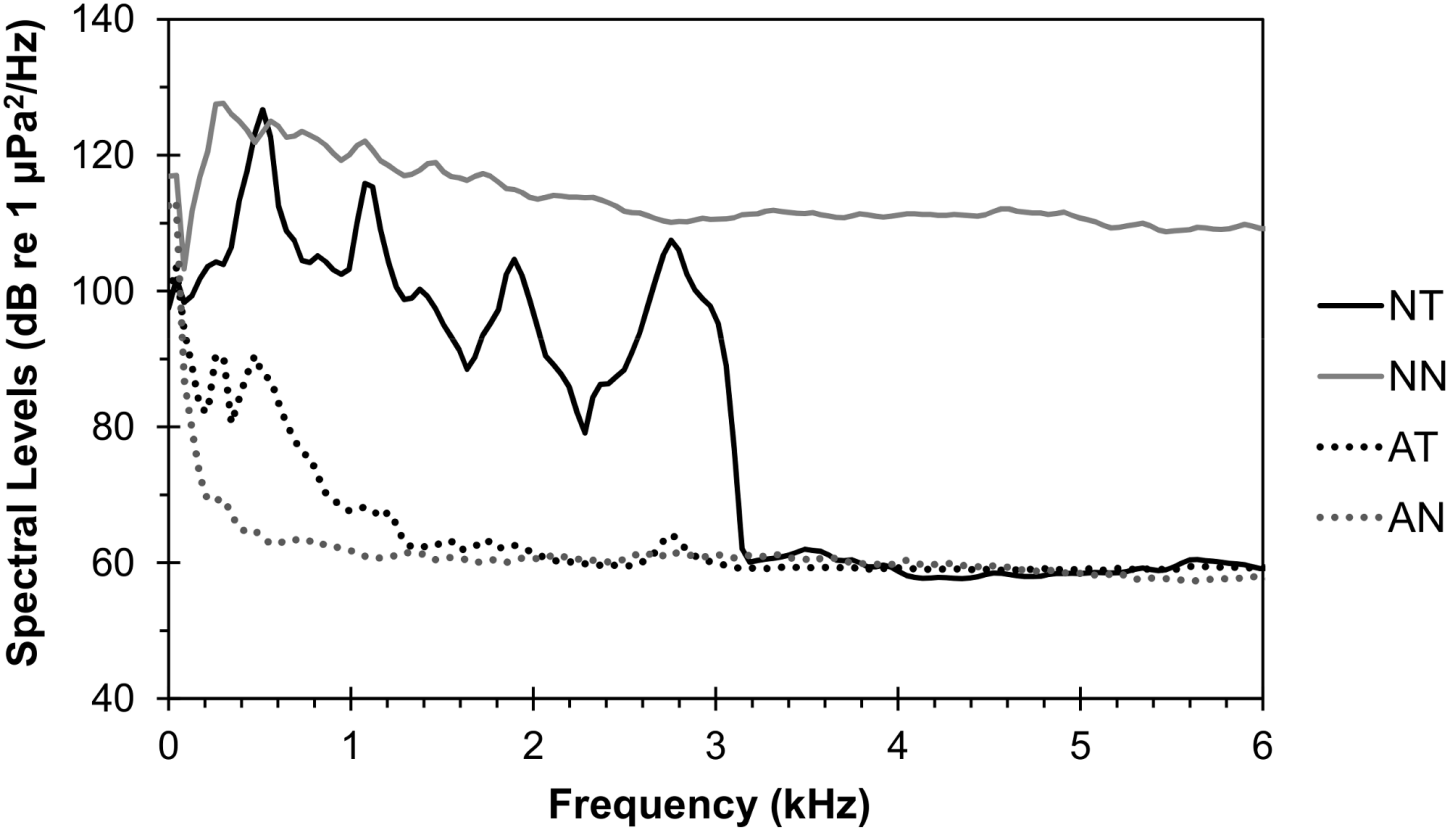
Average spectral levels of acoustic conditions in the experimental tank. Sound pressure levels of averaged power spectra (FFT spectrum level units normalised to 1 Hz bandwidth, Hann window, FFT size 1024, 50% overlap) of recordings during band-pass filtered additional-noise playbacks (0.1 to 3.0 kHz; NT) and control playbacks (AT) at two tank depths (5 cm above tank floor and 5 cm below water surface) at the location the fish had to be for the visual predatory stimulus to be released. For control playbacks, spectral levels from 30 s recordings were assessed and averaged over all playback tracks and the two tank depths; for additional-noise playbacks, spectral levels over the whole duration of single looped elements were taken, to account for power fluctuations within a recording of sound emitted by a moving ship, and averaged over all playback tracks and the two tank depths. Recordings were made with an omni-directional hydrophone with preamplifier (HTI 96-MIN; manufacturer-calibrated sensitivity −164.3 dB re 1 µPa; frequency range 2–30 000 Hz) and a solid-state recorder (Edirol R09HR, Roland Corporation), at a sampling frequency of 44.1 kHz and a sampling rate of 16 bits; recording levels calibrated against a 1 kHz reference tone of known amplitude. An example of original ship-noise (NN) and ambient-noise recording (AN) of a UK harbour are given for comparison.

Both control and additional-noise playback tracks were created from recordings made in the same three harbours: Plymouth (50°21′33″N, 4°7′26″W), Portsmouth (50°47′21″N, 1°6′25″W) and Gravesend (51°26′44″N, 0°22′0″E and 51°26′42″N, 0°22′37″E) (see [7], [14] for full details). Control tracks were based on nine ambient recordings when there were no boats or ships passing; additional-noise tracks from eight recordings when a single ship was passing. Since sound levels of ship-noise recordings peaked around 500 Hz (figure 1), additional-noise treatments likely overlapped with hearing ranges of both fish species (see Introduction; [46], [47]). Original recordings were band-pass filtered from 0.1 to 3.0 kHz (Avisoft: FFT 1024, Hann window). The lower boundary ensured that noises were only played within the effective frequency range of the underwater loudspeaker and the upper boundary was chosen to reduce sound resonances in the tank within the potential hearing range of the study species. Each filtered noise file was looped together to form a continuous playback track (30 min total for control tracks; 15 min total for additional-noise tracks); each additional-noise playback track contained noise generated from only one passing ship. Additional-noise tracks included 20 s fade in and out, from and to zero amplitude at a continuous rate, to avoid sudden onsets of noise and to simulate a ship approaching and leaving; control tracks included 10 s fade in and out (the shorter period was because maximum amplitudes were much lower than for additional-noise tracks; figure 1). Additional-noise track amplitudes and amplitudes between playback tracks from different original ship-noise samples were adjusted as described in Voellmy et al. [14].

During experimental trials, sounds were played back as wav files through a player (LOGIK 2GMP309; frequency ranges 20–20,000 Hz), amplifier (Kemo Electronic GmbH; 18 W; frequency response range: ∼40–20,000 Hz), potentiometer (set to minimum resistance; Omeg Ltd; 10 k logarithmic), and Aqua30 underwater loudspeaker (DNH; effective frequency range 80–20,000 Hz), as per Purser and Radford [53], Voellmy et al. [14], and Wale et al. [7], [28]. Individual playback tracks were used 2–5 times for a particular species to minimise pseudoreplication.

**Figure 2:**
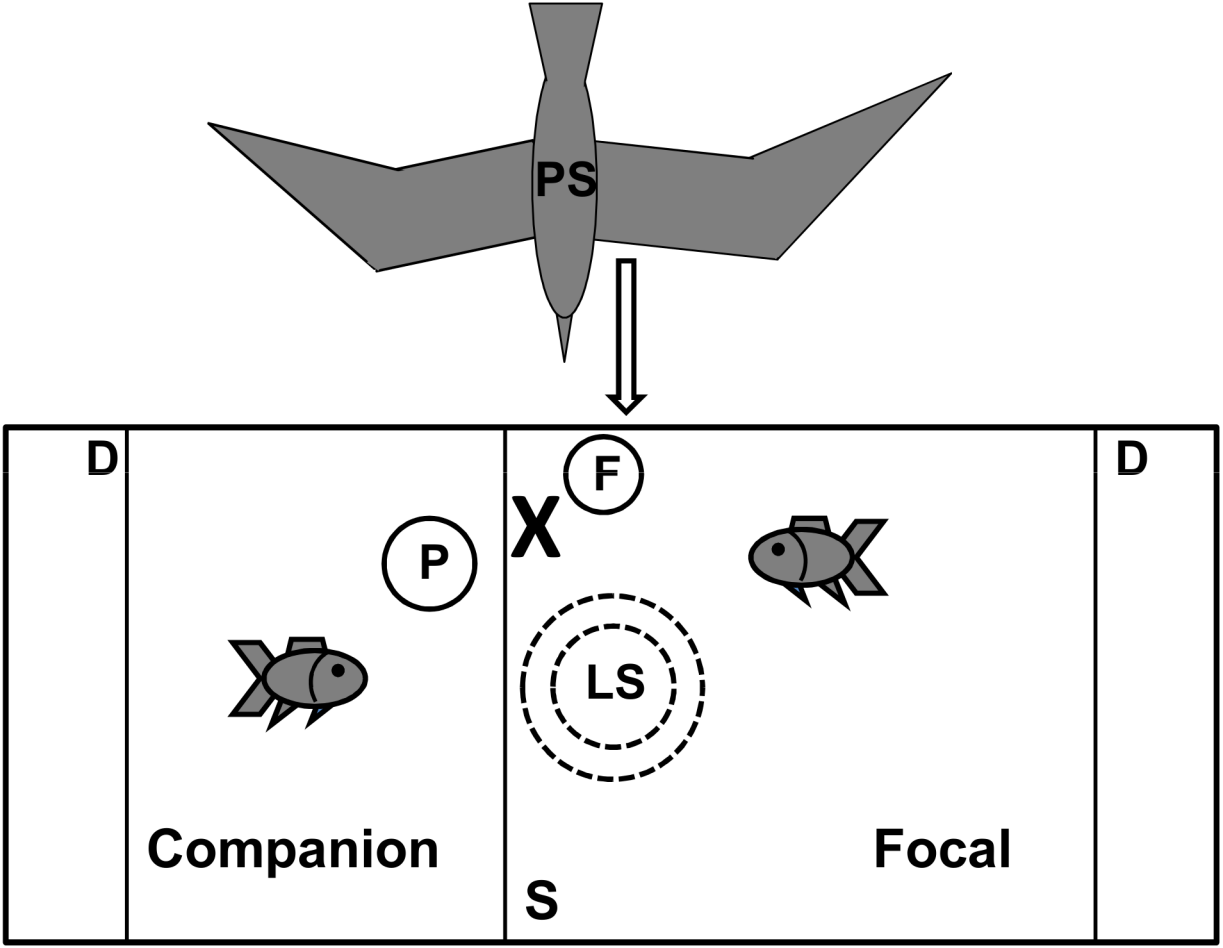
Overhead view of experimental tank setup. Schematic representation of visual predatory stimulus (PS), underwater loudspeaker (LS), focal fish position for predator release (X), feeder (F), artificial plant (P), mesh separator (S) and opaque Correx dividers (D).

The experiment was conducted in a 150×30 cm glass tank (water depth: 25 cm; wall thickness: 4 mm), with an upwards-facing underwater loudspeaker placed in the centre beneath a false 4 mm thick Correx floor positioned 10 cm above the bottom. The experimental tank was placed on three layers of 5 cm polystyrene pads (20×20 cm) and four layers of neoprene pads (20×20 cm) at six locations (each tank corner and two along the central line of the tank) along a laboratory side wall in a room separated from the main University building (to reduce various potential sources of noise; see [14]). Opaque Correx dividers (width: 4 mm) were placed 15 cm away from either end of the tank to minimise the influence of acoustic edge effects to the experimental area [30], [31]. Acoustic conditions during playbacks (in terms of sound pressure; figure 1) were measured at two tank depths (5 cm above tank floor and 5 cm below water surface) at the location where the fish had to be for the visual predatory stimulus to be released (see figure 2).

### Experimental protocol

Experimental trials were conducted between 8∶00 and 18∶30 when animals are active. The experiment involved exposing fish to a seagull model that ‘flew’ over the top of the tank (figure 2) during either control or additional-noise playbacks. The predatory model was mounted above the test tank with four nylon strings to allow ‘flight’ across the tank, perpendicular to the tank length, just above the focal fish section; this simulated a piscivorous bird shortly before diving and chasing after a prey fish. The model was attached on the laboratory wall using an additional nylon string and a cable roller to control its movement. Each focal fish received two trials (one for each sound treatment), separated by at least 30 min, randomly assigned to a trial series in counterbalanced order. A repeated-measures design was used to account for potential individual differences [54].

Prior to an experimental trial, a companion/focal fish pair of the same species were transferred to two adjacent sections of the test tank, using a net and two opaque jugs (one for each fish). The tank section for the companion fish contained an artificial plastic plant as shelter, the tank section for the focal fish a plastic feeder for guidance to the position where the focal fish had to be to release the predator stimulus (figure 2). Tank sections and positions of the mesh separator, plastic plant and feeder were alternated for experiments to different focal fish, but were kept the same for trials to the same fish. Fish were left to settle during playback of an ambient sound track until they resumed swimming and social interaction behaviour, and exhibited no hiding, startling, freezing or rapid escape attempts for 10 min.

After settling, playback was switched to either an additional-noise track or to a control track using an ambient sound track from the same location as the additional-noise track used for that fish. The new track was played for at least 1 min, and until the focal fish was half a fish length away from and perpendicular to the mesh separator and 1–2 fish lengths from the tank wall where the seagull was mounted. The seagull was then released and the response of the focal animal digitally video-recorded (Sony Handycam HDR-XR155E at 25 frames per second). Order of playback presentation was counterbalanced between fish of the same species. Between the two trials to the same animal, while the model was brought back to its original position, the fish were placed in two separate opaque measuring jugs. The fish were then returned to their respective tank sections and the experimental procedure repeated. From the videos, watched with muted sound and randomly assigned identification numbers, a single observer (IKV) recorded whether the fish responded (startled or froze) to the seagull presentation, and the response latency (time in seconds from release of the predatory stimulus to first response). These data are provided in Data File S1.

### Statistical analysis

Data were analysed in R version 2.15.1 [55] using mixed models to control for repeated testing of the same individual. In all analyses, stepwise backwards model simplification was used to determine the minimal model, with significance of model terms assessed by change in deviance upon removal of terms (ANOVA model comparison, Chi-squared test). Full (starting) models contained species (stickleback, minnow), noise treatment (control, additional) and individual’s trial order (control then additional, additional then control) as fixed factors, plus all two-way and three-way interactions of fixed factors, and subject as a random factor. The likelihood of responding to the visual predatory stimulus (response, no response), was modelled using a generalised linear mixed model (GLMM) with binomial error distribution and logit link function using the glmer function, lme4 package [56]; fitted by Laplace approximation. Latency to respond was modelled using mixed model Cox proportional hazards regression (MMCoxPH) with non-responders given maximum sampled latency and labelled as right-censored using the Surv function in survival package [57]; effects modelled using coxme function, coxme package [58]; fitted by maximum likelihood. Odds ratios of effects with 95% confidence intervals (CI) were calculated using the fixef function in lme4 package; assessed from minimal model, with term of interest added to minimal model when assessing non-significant effect. All quoted *p*-values are two-tailed and results were deemed significant at an alpha value of 0.05.

## Results

Sticklebacks (n = 35) were significantly more likely than minnows to respond to a visual predatory stimulus (GLMM: χ^2^_1_ = 18.0, p<0.001; stickleback odds 6.41 times higher than minnows (n = 27; CI: 2.63, 15.63)). However, there was no significant influence of noise treatment (χ^2^_1_ = 0.05, p = 0.828; additional-noise treatment odds 0.91 times that of control treatment (CI: 0.39, 2.13)). There was no significant order effect (χ^2^_1_ = 0.67, p = 0.414) and no significant effect of interactions between factors (treatment:order:species χ^2^_1_ = 0.01, p = 0.920; order:species χ^2^_1_ = 0.04, p = 0.849; treatment:order χ^2^_1_ = 0.55, p = 0.458; treatment:species χ^2^_1_ = 2.35, p = 0.126) on the likelihood of responding.

**Figure 3:**
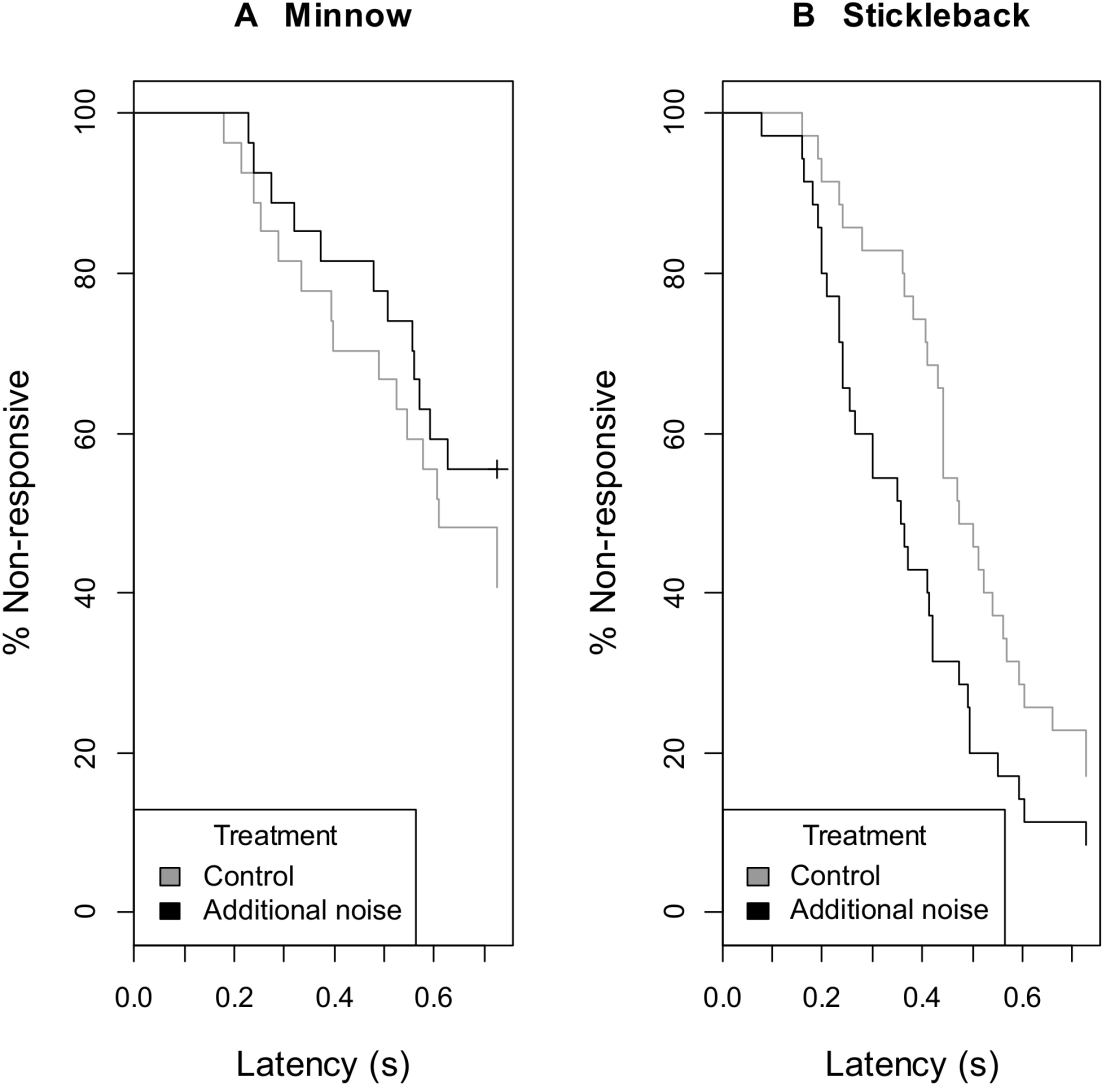
Speed of response to a visual predatory stimulus. Minnows showed no significant effect of noise treatment on response latency (A), while sticklebacks responded significantly more quickly during additional-noise playbacks compared to control playbacks (B). Plots of Kaplan-Meier estimate from mixed model Cox proportional hazards regression, with non-responders included as right-censored maximum-latency data. N = two trials to each of 27 minnows and 35 sticklebacks.

The effect of noise treatment on response latency significantly differed depending on species (MMCoxPH: interaction species:treatment χ^2^_1_ = 5.83, p = 0.016; figure 3). Examining species in turn, minnows (n = 27) showed no significant influence of noise treatment (χ^2^_1_ = 1.40, p = 0.245; additional-noise treatment odds 0.63 times that of control treatment (CI: 0.29, 1.36)), while sticklebacks (n = 35) showed significantly shorter latencies to respond in the additional-noise treatment (χ^2^_1_ = 6.80, p = 0.009; additional-noise treatment odds 2.13 times that of control treatment (CI: 1.23, 3.67)). There was no significant effect of any other interactions (treatment:order:species χ^2^_1_<0.01, p = 0.945; order:species χ^2^_1_ = 0.01, p = 0.905; treatment:order χ^2^_1_ = 2.65, p = 0.104) nor of order (χ^2^_1_ = 1.02, p = 0.31) on response latency.

## Discussion

Our results show that elevated noise levels can affect responses to a predatory threat, as shown in previous studies on crabs [6], [7], and European eels (*Anguilla anguilla*) [8]. While we found no effect of additional-noise playbacks on the most obvious behavioural measure (whether the fish responded to the predatory stimulus), there was an impact on response latency. This more subtle effect parallels earlier work on foraging behaviour [53], where sticklebacks exposed to increased noise did not consume fewer prey items, but did make more foraging errors and spent more time engaged in other activities than during control playbacks (but see [14]). Our testing of two fish species in the same conditions provides the first evidence that additional noise could affect the anti-predator responses of sympatric species in different ways: whereas sticklebacks responded more quickly to the predatory threat when exposed to additional-noise playback, minnows did not significantly differ in their response latency depending on sound treatment.

The faster anti-predatory response of sticklebacks when exposed to additional noise could be the result of increased vigilance, as seen in previous studies of terrestrial vertebrates [59], [60]. Increased noise levels might have triggered a stress response [61], resulting in greater general alertness and vigilance [62]. Alternatively, prey might compensate for potential masking of auditory predatory cues [63] by relying more on the use of visual information [60], and thus detect threats sooner. A reduced latency to respond could directly benefit survival [53], [63], as demonstrated in guppies (*Poecilia reticulata*) [64]: individuals that were in a position to detect a model predator sooner, initiated flight responses earlier and were more likely to escape predation when confronted with a real predator. However, responding faster reduces time available for threat assessment and may lead to suboptimal decisions, such as premature flight responses to non-threatening situations. If fleeing individuals seek shelter, and do not emerge for some time, such ‘escapes’ not only result in unnecessary energy expenditure, but may also lead to lost opportunities for foraging or reproduction [65]. In turn, compensation for lost foraging time may carry costs of increased predation risk, if animals are forced to forage during times of greater predatory threat [66].

Our work adds to the growing body of empirical evidence that the same noise source might not affect species in the same way [12]–[14]. While sticklebacks responded faster to the predatory threat when there was additional noise, crabs [6], [7] and eels [8] showed greater response latencies to a simulated predatory attack; minnows in our study showed no significant difference in response time depending on noise treatment, but any trend was also for an increased latency (figure 3). Previous studies have suggested distraction as the underpinning mechanism for slower responses [6]–[8]; that is unlikely to be the case with sticklebacks in this study, thus, not only the response but the underlying mechanism may differ between species. The absence of any noise-related effect in minnows might be because they did not hear the sound (although that seems unlikely given that they potentially have better hearing than sticklebacks; see Introduction) or heard it but did not pay any attention. Alternatively, the lack of an effect might be linked to the reduced overall likelihood of responding compared to sticklebacks; minnows were more likely to be interacting with their companion fish prior to the predator release (pers. obs.) and may thus have paid less attention to the predator stimulus resulting in lower response rates and in the case of a response, to longer response latencies compared to sticklebacks. Interspecific differences in the effect of noise could arise from differences in hearing ability [19], vulnerability to stress [11] or anti-predator defences [5], [24], and might have consequences on relative survival [14].

Laboratory studies such as ours offer the advantages of carefully controlled conditions and detailed data collection, which enables tight interspecific comparisons and consideration of subtle effects [67], as described in previous studies [7], [14], [53]. However, care is of course needed when translating such results to real-world contexts, as captive playback studies represent an artificial scenario [14], [67]. For example, the loudspeaker does not have a linear response and thus changes the spectral quality of the played back sounds, the sound field in a tank is complex and results in a different balance between the sound pressure and particle motion components of sound, and particle motion values are especially high such that results pertain to the near field (see Introduction). A few field studies on different species from those considered here have indicated that real anthropogenic noise sources affect, for instance, the movement of free-swimming shoals (e.g. [68], [69]) and time budgets between different activities [70], so it is likely that elevated noise can have an impact in natural conditions. Moving forward, the ideal studies would utilise a combined approach: carefully controlled experimental manipulations investigating potential effects with direct fitness consequences, but in the wild with real noise sources and thus allowing the spatial scale of impact to be determined. What is clear from our work, demonstrating the potential for additional noise to compromise anti-predator behaviour in species-specific ways, is the need to continue addressing how noise pollution affects individuals, populations and communities in the aquatic environment.

## Supporting Information

Data File S1 **Anti-predator response data.** This file contains all data collected on occurrence of response and latency to respond to a visual predatory stimulus of all European minnows and three-spined sticklebacks included in the study.
(XLS)
